# Genotype Analysis of *ABCC1*, *NCF4* and *CBR3* Polymorphism and the Association With Childhood Acute Lymphoblastic Leukemia in Mexican Childhood Population

**DOI:** 10.3389/fphar.2020.616630

**Published:** 2021-02-02

**Authors:** Jesús Alonso Gándara-Mireles, Ismael Lares-Asseff, Elio Aarón Reyes Espinoza, Javier G. Blanco, Isaias Chairez Hernández, Lourdes Patricia Córdova Hurtado, Verónica Loera Castañeda, Leslie Patrón Romero, Cristina Venzor Sánchez, Hugo Payan Gándara, Dinora Arechiga Gurrola, Horacio Almanza Reyes

**Affiliations:** ^1^ Instituto Politécnico Nacional, CIIDIR-Unidad Durango, Durango, México; ^2^ Red Latinoamericana de Implementación y Validación de Guías Clínicas Farmacogenómicas (RELIVAF-CYTED), Madrid, Spain; ^3^ Centro Estatal de Cancerología, Durango, México; ^4^ School of Pharmacy and Pharmaceutical Sciences, University of Buffalo, The State University of New York, Albany, NY, United States; ^5^ Facultad de Medicina y Psicología de la Universidad Autónoma de Baja California, Tijuana, México

**Keywords:** genetic polymorphisms, ABCC1, NCF4, CBR3, association, leukemia

## Abstract

**Background:** The identification of genetic risk factors for Acute Lymphoblastic Leukemia (ALL), are increasingly urgent and necessary.

**Objective:** The purpose of this study is to determine the association of the genetic polymorphisms *ABCC1* rs3743527, *NCF4* rs1883112 and *CBR3* rs1056892 with ALL.

**Methods:** DNA samples were obtained in 71 children with ALL (from 2 to 18 years) and in 71 controls without ALL, to determine the polymorphisms by real-time polymerase chain reaction (qPCR), using specific TaqMan probes in a StepOne^®^ thermal cycler (Applied Biosystems, United States).

**Results:** The results of the Odds Ratio analysis show that in the rs1883112 polymorphism of the NCF4 gene, the heterozygous allele has a risk effect for ALL (OR = 3.1870, CI = 1.8880–7.9383 and *p* = 0.0002), in turn the mutated genotype (AA) is associated with a protective effect (OR = 0.26, 0.1248 to 0.5434 and *p* = 0.0003). On the other hand, the CBR3 rs1056892 polymorphism shows a significant association of risk to ALL, in the presence of the HT genotype (OR = 2.77, IC = 1.3837 to 5.5651 and *p* = 0.004) and the mutated genotype of this polymorphism has a significant association with protection to ALL in the HM genotype (OR = 0.52, IC = 0.2639 to 1.0304 and *p* = 0.05). While the inheritance models of the polymorphisms let us see that of the rs1883112 polymorphism of the NCF4 polymorphism; the HT genotype of the codominant model shows a protective effect against ALL (OR = 0.4117, IC = 0.1718 to 0.9866 and *p* = 0.04), the recessive model shows us and confirms what we already saw in table number 3, being that there is an association with protective effect in the HM genotype (OR = 0.2604, IC = 0.1248 to 0.5434 and *p* = 0.0003). In the polymorphism rs1056892 of the CBR3 gene, a protection association was found in the heterozygous allele of the codominant model (OR = 0.3448, IC = 0.1375 to 0.8896 and *p* = 0.0274). In addition, the recessive inheritance model for the HM genotype shows a protective effect to ALL, (OR = 0.52, CI = 0.9919 to 3.8638 and *p* = 0.05).

**Conclusion:** There is an evident impact of the NCF4 rs1883112 and CBR3 rs1056892 polymorphisms with an increased risk of susceptibility to ALL; Likewise, through the codominant inheritance model, the effect of the variation of the CBR3 rs1056892 gene as a protective factor against ALL was evaluated.

## Introduction

Childhood Acute lymphoblastic leukemia (ALL) is the most common pediatric cancer in developed countries and also it is a malignant disease of the white blood cells with a multifactorial etiology that likely involve an interplay of environmental and genetic variables. Is estimated that more than 60% of patients diagnosed with ALL are children below the age of 15 years, with a peak of incidence at 2–5 years of age. With the actual treatment scheme, the rate of cure is 90% (Rivera-Luna et al., 2005; Pérez-Saldívar et al., 2011; Secretaria de Salud et al., 2011). The implementation of risk-stratified therapy has been successful, as survival rates for ALL have improved significantly, with overall survival rates of 5 years currently (Rodriguez et al., 2010; Muwakkit et al., 2012; Ceppi et al., 2015; Frost et al., 2018). A wide and deep understanding of genetic factors, as well as chromosomal abnormalities, gene expression, response to therapeutic treatment, and host pharmacogenomics, offer the potential to improve prognosis and therapeutic optimization in the treatment of childhood ALL (Vrooman and Silverman, 2009). The Genome Wide Association Study (GWAS) provide a candidate genes and cellular pathways to understand the etiology, also to determine candidates’ genes to improve the treatment response and pharmacogenomics. These single nucleotide polymorphisms (SNP's) have been largely study over the last decades in ALL. Some of these pathways involve inflammation. Liu et al. (2020) in a case-control study found that polymorphisms of IL-6 and IL-10 are significantly correlated with the susceptibility and pathogenesis of ALL in childhood (Liu et al., 2020). Some other environmental factors associated with leukemiogenesis are maternal vitamins intake during pregnancy, particularity folic acid and its fetal metabolism. Folic acid (or vitamin B9) and its derivatives, collectively known as folates, are chemoprotective micronutrients of great interest belonging to the B vitamin group. Folic acid is involved in the correct production and maturation of blood cells from hematopoietic stem cells, DNA methylations, DNA synthesis and repair, proper gene expression, chromosomal and nuclear integrity. In particular, folic acid supplementation during pregnancy has a protective effect towards ALL, with a diminished incidence of 15–21% (Cantarella et al., 2017). Inside the cell, folic acid is transformed into tetrahydrofolate (THF), a reaction carried out by the enzyme NADPH oxidase-dependent hydrofolate reductase, this being a membrane-bound enzyme complex that faces the extracellular space (Navarro-Pérez et al., 2016). On the other hand, the neutrophil cytosolic factor 4 (NCF4) is a protein encoded in humans by the *NCF4* gene, which encodes the p40-phox (NADPH) oxidase subunit of the NOX2; this protein is part of the NADPH oxidase complex (Zhan et al., 1996). NOX2 complex has been related to at least ovarian cancer and renal cell carcinoma (Meitzler et al., 2014). Also, there is evidence that the NADPH-dependent oxidase NOX2 is an important effector of immune cell function, and its activity has been linked to oncogenic signaling (Adane et al., 2019). Gutiérrez-Salinas J. and collaborators (Gutiérrez-Salinas et al., 2016) in 2016 reported that oxidative stress promote the development of cancer, and carbonylated proteins may have a role in the carcinogenesis process. Carbonyl reductase three encoded by the *CBR3* gene catalyzes the reduction of a large number of biologically and pharmacologically active carbonyl compounds; a polymorphism present in this gene, such as rs1056892, may have implications for the risk of developing ALL. Other pathway involved in the overcome, prognostic and surveillance is the drug response to the treatment. One of the most important gene in this field is the *ABCC1* gene. Multidrug resistance-associated protein 1 (MRP1), encoded by the *ABCC1* gene, is an ATP-binding cassette transporter mediating efflux of organic anions and xenobiotics; its overexpression leads to multidrug resistance. Many drugs are good substrates for MRP1, so its overexpression leads to multidrug resistance (MDR), especially during cancer chemotherapy. This effect can be observed in many types of cancer cells including solid tumors (lung cancer, breast cancer, gastric and colon carcinomas, melanoma, prostate cancer, neuroblastoma) as well as in various types of leukemias. There exist ethnic differences in the frequency of *ABCC1* polymorphic variants and least 95 SNPs have been described in different population with also wide diversity (Słomka et al., 2016). Due to the importance of identifying risk factors for developing ALL, the objective of the present study is to determine if the *ABCC1* rs3743527, *NCF4* rs1883112 and *CBR3* rs1056892 genetic polymorphisms are associated with susceptibility to ALL in pediatric population. Therefore, we investigated the association between these three SNPs involved in the metabolism of antineoplastic drugs and ALL susceptibility.

## Methods

The Research Ethics Committee and the Research Committee of the General Hospital of Durango Torre Materno-Infantil, México, approved and validated the study according to the declaration of Helsinki and the General Health Law of Mexico. 71 pediatric patients of both sexes treated at the Pediatric Hemato-oncology Service of the State Cancer Center (CECAN) of the Secretary of Health of Durango, Mexico were studied. All patients were diagnosed with ALL according to the criteria of the Franco-American-British Hematology Association (Bennett et al., 1976). Each patient was undergoing chemotherapy treatment according to the St Jude TOTAL XV (Pui et al., 2010) protocol. Patients were also included in surveillance, that is, children who had completed their pharmacological treatment. In addition, a group of 71 adolescents without ALL was evaluated as a control group. All parents of patients were asked to sign the informed consent, in addition to children over 8 years of age, they were asked to accept the informed consent.

### Genotypification

DNA was obtained from whole blood using the extraction procedure “DTAB-CTAB” (Gustincich et al., 1991), its integrity and purity was determined by horizontal electrophoresis in 1% agarose gel, stained with Texas Red and the quantification was carried out by spectrophotometry in Nanodrop^®^ (Thermo Scientific, United States). The SNPs of the *ABCC1* rs3743527 gene (Probe number: C___8934057_30), the *NCF4* rs1883112 gene (Probe number: C__11521119_1_) and the *CBR3* rs1056892 gene (Probe number: C___9483603_10) were determined by qPCR using specific TaqMan probes for each polymorphism (by Thermo Fisher Scientific^®^), in a StepOne^®^ thermocycler.

### Statistic Analysis

The genotypic and allelic frequencies of the two groups were obtained; with ALL (cases) and without ALL (control). In addition, the Hardy-Weinberg equilibrium analysis (HWE) was performed. The software used was the SNPStats (Catalan Institute of Oncology, Barcelona, Spain) (Solé et al., 2006). Subsequently, the frequency of the alleles between the cases and the controls of each polymorphism was compared using the Chi square test (*x* ^2^), a value of *p* < 0.05 was considered statistically significant. Finally, an Odds Ratio (OR) (McHugh, 2009) risk association analysis was performed for each allele of each of the polymorphisms between the cases (with ALL) and the controls (without ALL), as well as the inheritance models in which a value *p* <0.05 was considered statistically significant.

## Results

Table 1 shows the demographic data for the two groups, patients with ALL (cases) and without ALL (controls), with a statistically significant difference between the biological variables considered, except for age (*p* = 0.09).

**TABLE 1:** Demographic aspects of patients with ALL (cases) and volunteers without ALL (controls).

Variables	Cases (*n* = 71)	Controls =71	*p*
Age (years)	13.2 ± 4.8	14.1 ± 3.1	0.09
Gender (♂/♀)	45/22	28/39	
Weight (Kg)	32.2 ± 15.99	64.7 ± 13.2	<0.0005
Height (cm)	123.7 ± 26.4	164.0 ± 22.3	<0.0005
BMI (Kg/m^2^)	18.5 ± 3.2	23.1 ± 3.5	<0.0005

^a^ T Student test.

Table 2 shows the genotypic and allelic frequencies of patients with ALL for each polymorphism. Also as is shown, the NCF4 rs1883112 polymorphism does not find Hardy Weingber Equilibrium (EHW), while the CBR3 rs1056892 and ABCC1 rs374352 polymorphisms do.

**TABLE 2:** Genotypic and allelic frequencies of *NCF4* rs1883112, *CBR3* rs1056892 and *ABCC1*. rs3743527 polymorphisms in cases and controls in children with ALL.

Polymorphisms		Cases (*n* = 71)	Controls (=71)	*p* ^a^		HWE^b^
	Genotype WT (GG	17 (24%)	18(25%)			
	HT (AG)	39(55%)	17(24%)	0.02	No	
*NCF4* rs1883112	HM (AA) Allelos	15 (21%)	36 (51%)			
	Major allele (G)	73(52%)	53(37%)			
	Minor allele (A)	69(48%)	89(63%)			
	Genotype WT (AA)	11(15%)	17(24%)			
	HT (AG)	37(52%)	20(28%)	0.15	Yes	
*CBR3* rs1056892	HM(GG) Allele	23 (33%)	34 (48%)			
	Major allele (G)	83(59%)	88(62%)			
	Minor allele (A)	59 (41%)	54 (38%)			
	Genotype WT (CC)	31(44%)	26(37%)			
	HT (CT)	32(45%)	41(58%)	0.25	Yes	
*ABCC1*	HM (TT)	8 (11%)	4 (5%)			
rs3743527	Allele					
	Major allele (C)	92(66%)	93(66%)			
	Minor allele (T)	48(34%)	49(34%)			

^a^ Chi square test.

^b^ HWE (Hardy Weinberg Equilibrium).

Table 3 shows the association of WT, HT and HM genotypes with ALL through Odds Ratio values for each polymorphism. In the rs1883112 polymorphism of the *NCF4* gene, it is observed that the heterozygous allele has a risk effect for ALL (OR = 3.1870, CI = 1.8880 to 7.9383 and *p* = 0.0002), in turn the mutated genotype is associated with a protective effect (OR = 0.26, 0.1248 to 0.5434 and *p* = 0.0003). On the other hand, the *CBR3* rs1056892 polymorphism shows a significant association of risk to ALL, in the presence of the HT genotype (OR = 2.77, IC = 1.3837 and *p* = 0.004) and the mutated genotype of this polymorphism has a significant association with protection to ALL in the HM genotype (OR = 0.52, IC = 0.2639 to 1.0304 and *p* = 0.05). A significant association for any genotype in the *ABCC1* rs3743527 polymorphism was not found. Finally, the inheritance models of the studied polymorphisms are shown (Table 4) where it is observed that in the case of the rs1883112 polymorphism of the *NCF4* gene; the HT genotype of the codominant model shows a protective effect to ALL (OR = 0.4117, IC = 0.1718 to 0.9866 and *p* = 0.04), the recessive model shows us and confirms what we already saw in table number 3, since there is an association with protective effect on the HM genotype (OR = 0.2604, IC = 0.1248 to 0.5434 and *p* = 0.0003). In the rs1056892 polymorphism of the *CBR3* gene, a protective association was found in the heterozygous allele of the codominant model (OR = 0.3448, CI = 0.1375–0.8896 and *p* = 0.0274), in addition, the recessive inheritance model for the HM genotype shows a protective effect to ALL (OR = 0.52, IC = 0.9919 to 3.8638 and *p* = 0.05). Regarding *ABCC1* rs3743527, no statistically significant associations were found for ALL.

**TABLE 3:** Estimation of risks by Odds Ratio between cases and controls of polymorphisms *NCF4* rs1883112, *CBR3* rs1056892 and *ABCC1* rs3743527.

Polymorphisms	Allele	Cases	Controls	OR	*IC*	*p*
	WT	17	18			
				0.92	0.4320 to 1.9892	0.84
	HM + HT	54	53			
*NCF4rs1883112*	HT	39	17	3.87	1.8880 to 7.9383	0.0002
	HM + WT	32	54			
	HM	15	36			
				0.26	0.1248 to 0.5434	0.0003
	WT + HT	56	35			
	WT	11	17	0.58	0.2507 to 1.3528	0.20
	HM + HT	60	54			
	HT	37	20			
*CBR3rs1056892*				2.77	1.3837 to 5.5651	0.004
	HM + WT	34	51			
	HM	23	34			
				0.52	0.2639 to 1.0304	0.05
	WT + HT	49	37			
	WT	31	26			
				1.34	0,6842 to 2,6295	0.39
	HM + HT	40	45			
	HT	32	41			
*ABCC1rs3743527*				0.60	0.3092 to 1.1657	0.13
	HM + WT	39	30			
	HM	08	04			
				2.12	0.6103 to 7.4133	0.23
	WT + HT	63	67			

**TABLE 4:** Association by models of inheritance of polymorphisms *NCF4* rs1883112, *CBR3*. rs1056892 and *ABCC1* rs3743527 at risk of developing LLA.

Polymorphisms		Cases(*n* = 71)	Controls(*n* = 71)	OR	CI	*p*
	Codominant					
	WT (GG)	17	18	1		
	HT (GA)	39	17	0.4117	0.1718 to 0.9866	0.04
	HM (AA)	15	36	2.2667	0.9256 to 5.5510	0.07
	ALLELE (G)	73	53			
*NCF4* rs1883112	ALLELE (A) Dominant	69	89	1.7766	1.1068 to 2.8517	0.01
	WT	17	18			
	HT + HT	54	53	0.9285	0.4320 to 1.9892	0.84
	Recessive					
	HM	15	36			
	WT + HT	56	35	0.2604	0.1248 to 0.5434	0.0003
	Codominant WT (AA)	11	17	1		
	HT (AG)	37	20	0.3448	0.1375 to 0.8896	0.0274
	HM (GG)	23	34	0.4565	0.1611 to 2.2062	0.9249
*CBR3*	ALLELE (A)	59	54			
rs1056892	ALLELE (G)	83	88	0.3686	0.2094 to 0.6488	0.5445
	Dominant					
	WT	11	17	0.5824	0.2507 to 1.3528	0.2086
	HT + HM	60	54			
	Recessive					
	HM	23	34	0.5200	0.9919 to 3.8638	0.05
	WT + HT	49	37			
	Codominant WT (CC)	31	26	1		
	HT (CT)	32	41	1.5276	0.7611 to 3.0662	0.2333
	HM (CT)	08	04	0.5962	0.1611 to 2.2062	0.4385
*ABCC1*	ALLELE (C)	94	93			
r3743527	ALLELE (T)	48	49	1.0318	0.6318 to 1.6851	0.9004
	Dominant					
	WT	31	26	1.3413	0.6842 to 2.6295	0.3925
	HT + HM	40	45			
	Recessive					
	HM	08	04	2.1213	0.6103 to 7.4133	0.2361
	WT + HT	63	67			

**FIGURE 1:**
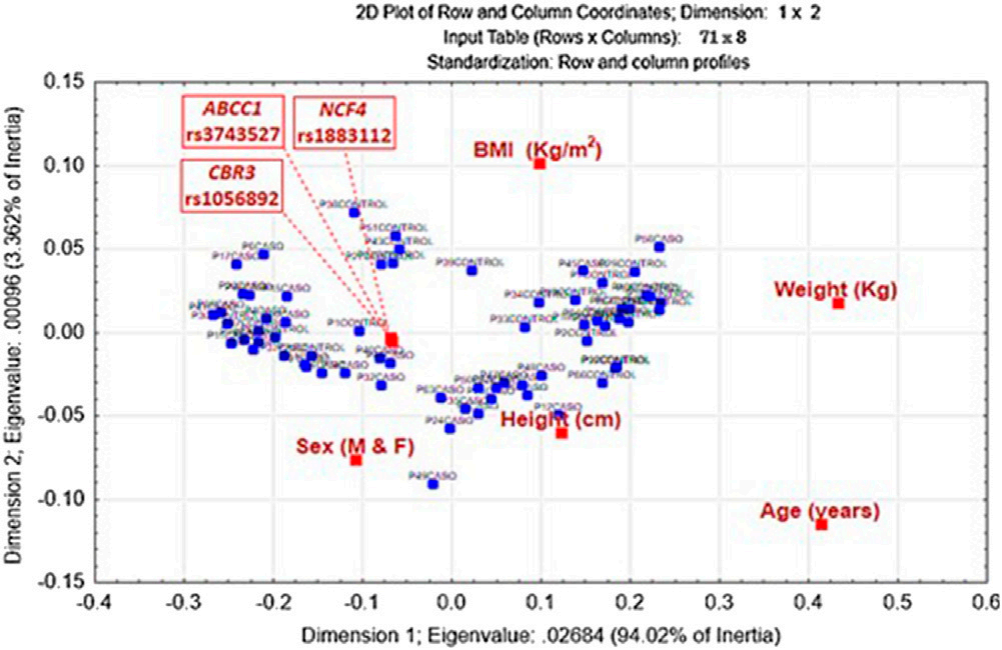
Association of the eight variables (red box) with patients with All (blue box). The relative inertia of each variable shows that; Height (Relative Inertia: 0.610400), *NCF*4 rs1883112 polymorphism (Relative inertia: 0.067791), and Sex (Relative Inertia: 0.058756) had a greater association with the development of ALL.

Figure 1 shows the multivariate analysis using the correspondence test, in this analysis it can be observed that the variables that had the greatest association with the development of ALL were 1)- height, 2)- *NCF4* rs1883112 polymorphism, and 3)- sex. The relative inertia values that indicate the level of association of each variable with the patients with ALL are shown in Table 5.

**TABLE 5:** Relative inertias of each variable associated with the development of ALL.

	Relative inertia	*p*
Age	0.013539	0.0001^a^
Sex	0.058756	
Height	0.610400	
Weight	0.002540	
BMI	0.037701	
NCF4 rs1883112	0.067791	
CBR3 rs1056892	0.057491	
ABCC1 rs3743527	0.055791	

^a^ Chi square test

## Discussion

There are several factors that are involved in the development of ALL in pediatric patients, such factors can be chemical, environmental and genetic (Steinberg et al., 2007; Cangerana-Pereira et al., 2017), these factors can modify gene expression. Many of these genes are involved in the metabolism and transport of certain anti-leukemic drugs and may also be directly or indirectly involved in the development of cancer, as in the case of NADPH-dependent enzymes (Minotti et al., 2000; Blanco et al., 2012) and/or cellular transporters. Other cellular transporters are the ABCC1 superfamily (Zaruma-Torres et al., 2015) constituted by 48 structurally similar membrane transporters. Variants in *ABC* genes that affect gene function have clinically important effects on drug disposition and can be predictors of the risk of adverse drug reactions and efficacy of chemotherapeutics, calcium channel blockers, and protease inhibitors (Xiao et al., 2020). Kunická T. and Souček P., (Kunická and Souček 2014) mentioned that resistance to multiple drugs has one of the most important causes of cancer treatment failure, ABC transporters are membrane-bound proteins that participate in cell defense mechanisms, hence their function is to prevent toxicity as carcinogenesis on the one hand, but may contribute to resistance of tumor cells to a number of drugs, including chemotherapeutics. Zaruma-Torres F et al. (Zaruma-Torres et al., 2016) in 2016 found that the *ABCB1* rs1045642 and *ABCC5* rs3792585 polymorphisms were associated with an increased risk of ALL in Mexican children. In the current study, we determinate the impact of three gene polymorphisms involved in the metabolism of antineoplastic drugs, such as the *NCF4* rs1883112, *CBR*3 rs1056892 and *ABCC1* rs3743527 polymorphisms, we also provided frequencies of this variants and identify any association with the development of ALL in children. In 2016 Gutierrez-Salinas Torres et al. (Gutiérrez-Salinas et al., 2016), determined the concentrations of carbonylated proteins and the carbonyl reductase enzyme in Mexican women with breast cancer, finding that oxidative stress could promote cancer development and involve carbonylated proteins in the process carcinogenic, with a 3.76-fold increase in plasma carbonylated proteins in the patient group, compared to the healthy control group (5 ± 3.27 vs. 1.33 ± 2.31 nmol carbonyls/mg protein; *p* < 0.05); In addition, they found a 60% increase in the carbonyl reductase enzyme in the patients vs the control group (3.27 ± 0.124 vs. 2.04 ± 0.11 ng/mg protein; *p* <0.05), a positive correlation was also found (*r* = 0.95; *p* <0.001) between both measurements, which may suggest the presence of tissue damage caused by cancer. On the other hand, Osawa Y et al. (Osawa et al., 2015) had previously reported the importance of the expression levels of an enzyme of the “CBR” family, finding that the expression level of carbonyl reductase (CBR1) is related to tumor progression. Decreased CBR1 expression is associated with a poor prognosis in ovarian cancer; they also investigated the relationship between the level of CBR1 expression and the malignant potential of ovarian cancer. These results showed that the decrease in CBR1 promoted tumor proliferation and growth, as well as invasion and metastasis, suggesting that CBR1 has the potential to become a new candidate for molecular targeting therapy. Unlike the previous results, in our study, the importance of a polymorphism that modifies the sequence of the “CBR” family gene is reflected, since it was found that for the *CBR3* rs1056892 polymorphism there is a significant association of risk to ALL in presence of the HT genotype (OR = 2.7750 (IC = 1.3857–5.5651), *p* = 0.004), however, when analyzing the inheritance models we found that in the case of the codominant inheritance model, the heterozygous allele of the *CBR3* gene constitutes a protection factor according to the obtained values (OR = 0.3448 (IC = 1.375 to 0.8896), *p* = 0.02). It is evident that, although there are many factors that are involved in the development of leukemia, genetic factors play a very important role since within the genes that are involved in different cellular metabolic pathways, some of them seem to have more importance. Apparently, the folate route constitutes a very important pathway, such is the case of the work reported by Zaruma-Torres F et al. in 2016 (Zaruma-Torres et al., 2016) who determined the associations between six SNPs in four genes related to the folate transporter pathway, to determine a significant relationship with the appearance of ALL in Mexican children, concluding that certain genetic polymorphisms related to the folate transport route, particularly *COL18A1* rs2274808, *SLC19A1* rs2838956, *ABCB1* rs1045642 and *ABCC5* rs3792585, were associated with an increased risk of ALL in Mexican children. These results coincide, although to a lesser extent with what was found in our work, in which other polymorphisms are related to an increased susceptibility to ALL, such as the case of *NCF4* rs1883112, became evident since this factor NCF4 could influence the susceptibility of presenting ALL through this polymorphism, as it hinder the action of folic acid and promote the risk of developing ALL, possibly by making it difficult to transform the folic acid to THF, through the de-hydro folate reductase dDHFR which is dependent on NADPH oxidase. There is not experimental data to demonstrate its functional significance, however the rs1883112 polymorphism is found in the NCF4 promoter (p40phox), which participates in the negative regulation of NADPH oxidase (Zhang et al., 1996). In the study by Lopes LR (Lopez et al., 2004), they demonstrated that the addition of phosphorylated p40 (PHOX) to the cell-free system inhibits the NADPH oxidase activated by the C-phosphorylated protein kinase p47 (PHOX), an effect not observed with non-phosphorylated p40 (PHOX). Furthermore, phosphorylated p40 (PHOX) inhibits oxidase if added before or after full activation of the enzyme, thus they have postulated that phosphorylation of p40 (PHOX) in threonine 154 leads to an inhibitory conformation that changes the balance towards an inhibitory role and blocks oxidase activation in such a way that in our study we found that for the rs1883112 polymorphism of the *NCF4* gene, the heterozygous allele shows a risk effect for ALL (OR = 2.1267 (CI = 1.0020–4.5138), *p* = 0.04. The Mexican population has a great ethnic diversity with 68 indigenous groups (International Work Group for Indigenous Affairs, 2020). The presence of genetic bias is evidenced or ruled out through the analysis of the Hardy-Weinberg equilibrium test (EHW), in the present study it is observed as two of the three Polymorphisms are in equilibrium, however one is not in equilibrium with a predominance of the mutated allele in the control group, which was carefully selected not to include genetically related people, which suggests that perhaps there is a predominance of this mutated allele in the population studied for a possible founder effect.

## Conclusion

In clinical practice, we can point out that a patient with an HT genotype for the polymorphisms of the CBR3 rs1056892 and NCF4 rs1883112 genes will have a higher risk of developing ALL. For the NCF4 rs1883112 gene polymorphism, the mutated allele will itself have an ALL risk effect, but two copies of it will not be additive; therefore, it is necessary for said mutated allele to bind to a copy of the wild allele (this being a heterozygous genotype) to consider it as a risk factor for ALL.

For the polymorphisms of the NCF4 rs1883112 and CBR3 rs1056892 genes, the codominant model shows us that both alleles (wild and mutated) have an association with a separate protective effect, even having two copies of these alleles still gives rise to a protective association against the ALL.

## Data Availability Statement

The original contributions presented in the study are included in the article/Supplementary Material, further inquiries can be directed to the corresponding author.

## Ethics Statement

The studies involving human participants were reviewed and approved by Comité de Ética en Investigación del Hospital General Materno Infantil de Durango, México. Written informed consent to participate in this study was provided by the participants’ legal guardian/next of kin.

## Author Contributions

Conception: IL-A. Interpretation or analysis of data: JG-M, IL-A, JB, and VL. Preparation of the manuscript: JG-M, IL-A, JB, EE, LH, HR, LR, CS, HG, and DG. Revision for important intellectual content: IL-A, JB, and HR. Supervision: IL-A, EE, and JB.

## Funding

To the Instituto Politécnico Nacional for project support: SIP 20200634.

## Conflict of Interest

The authors declare that the research was conducted in the absence of any commercial or financial relationships that could be construed as a potential conflict of interest.
